# Prenatal exposure to ambient fine particulate matter and child lung function in the CANDLE cohort

**DOI:** 10.1080/07853890.2024.2422051

**Published:** 2024-11-04

**Authors:** Allison R. Sherris, Marnie F. Hazlehurst, Logan C. Dearborn, Christine T. Loftus, Adam A. Szpiro, Margaret A. Adgent, Kecia N. Carroll, Drew B. Day, Kaja Z. LeWinn, Yu Ni, Sheela Sathyanarayana, Rosalind J. Wright, Qi Zhao, Catherine J. Karr, Paul E. Moore

**Affiliations:** a Department of Environmental and Occupational Health Sciences, School of Public Health, University of Washington, Seattle, WA, USA; b Department of Biostatistics, School of Public Health, University of Washington, Seattle, WA, USA; c Department of Health Policy, Vanderbilt University Medical Center, Nashville, TN, USA; d Department of Pediatrics, Department of Environmental Medicine & Public Health, Icahn School of Medicine at Mount Sinai, New York, NY, USA; e Department of Child Health, Behavior, and Development, Seattle Children’s Research Institute, Seattle, WA, USA; f Department of Psychiatry and Behavioral Sciences, University of California, San Francisco, CA, USA; g School of Public Health, College of Health and Human Services, San Diego State University, San Diego, CA, USA; h Department of Pediatrics, School of Medicine, University of Washington, Seattle, WA, USA; i Department of Environmental Medicine and Climate Science, Institute for Climate Change, Environmental Health, and Exposomics, Icahn School of Medicine at Mount Sinai, New York, NY, USA; j The University of Tennessee Health Science Center, Memphis, TN, USA; k Division of Allergy, Immunology, and Pulmonary Medicine, Department of Pediatrics, Vanderbilt University Medical Center, Nashville, TN, USA

**Keywords:** PM2.5, air pollution, airway, children’s health

## Abstract

**Background:**

Ambient fine particulate matter (PM_2.5_) exposure adversely impacts child airway health; however, research on prenatal PM_2.5_ exposure, and child lung function is limited. We investigated these associations in the ECHO-PATHWAYS Consortium, focusing on the role of exposure timing during different phases of fetal lung development.

**Methods:**

We included 675 children in the CANDLE cohort born between 2007 and 2011 in Memphis, TN, USA. Prenatal exposure to ambient PM_2.5_ was estimated using a spatiotemporal model based on maternal residential history and averaged over established prenatal periods of lung development. Forced expiratory volume in the first second (FEV1) and forced vital capacity (FVC) were measured by spirometry at age 8–9 years. We used linear regression and Bayesian Distributed Lag Interaction Models (BDLIM) to estimate associations between exposure and lung function z-scores, adjusting for maternal/child characteristics, prenatal/postnatal tobacco exposure, and birth year/season, and evaluating effect modification by child sex and allergic sensitization.

**Results:**

The average ambient concentration of PM_2.5_ during pregnancy was 11.1 µg/m^3^ (standard deviation:1.0 µg/m^3^). In the adjusted linear regression and BDLIM models, adverse, but not statistically significant, associations were observed between exposure during the pseudoglandular (5–16 weeks of gestation) and saccular (24–36 weeks) phases of lung development and FEV1 and FVC. The strongest association was between a 2 μg/m^3^ higher concentration of PM_2.5_ during the saccular phase and FEV1 z-score (−0.176, 95% Confidence Interval [CI]: −0.361, 0.010). The FEV1/FVC ratio was not associated with PM_2.5_ in any exposure window. No effect modification by child sex or allergic sensitization was observed.

**Conclusions:**

We did not find strong evidence of associations between prenatal ambient PM_2.5_ exposure and child lung function in a large, well-characterized study sample. However, there was a suggested adverse association between FEV1 and exposure during late pregnancy. The saccular phase of lung development might be an important window for exposure to PM_2.5_.

## 1. Introduction

There is substantial evidence that exposure to air pollution can affect a child lung function, an important indicator of airway disease and a determinant of future respiratory health [1–3]. Short- and long-term postnatal exposure to fine particulate matter less than 2.5 µm in diameter (PM_2.5_) has been consistently associated with decreased lung function in childhood [4,5]. However, the role of *in utero* exposure to PM_2.5_ remains poorly understood [3,5]. Limited studies have identified associations between prenatal PM_2.5_ exposure and lower forced expiratory volume in 1 s (FEV1; a measure of airway obstruction) and forced vital capacity (FVC; a proxy for lung volume) [6–9], while other studies have found no associations [10].

Lung development begins within the first week of gestation and continues throughout early childhood [11,12]. This period of rapid development may be sensitive to impacts from ambient pollutants including PM_2.5_ through pathways such as oxidative stress, inflammatory responses, or epigenetic programming [13–15]. Prenatal PM_2.5_ exposure has been associated with an increased risk of childhood asthma and respiratory symptoms [16,17], yet comparatively little research has been conducted on quantitative measures of child lung function. Furthermore, the potential critical windows of exposure to PM_2.5_ during fetal lung development are not clear.

Findings have also been mixed regarding sub-populations that may be more vulnerable to the airway impacts of prenatal PM_2.5_; one study found a stronger association between prenatal PM_2.5_ exposure and child lung function among boys [9], while another found no difference in associations by child sex [7]. Results for effect modification of the relationship between other air pollutants and lung function have also been mixed [18–20]. For example, studies have identified stronger associations between NO_2_ and lung function among children with allergic sensitization [18,19], though the role of allergic response in mediating or modifying the impact of prenatal exposure to PM_2.5_ is not well understood.

The CANDLE cohort offers the opportunity to explore the role of prenatal PM_2.5_ exposure and lung development in a large, socio-demographically diverse cohort with comprehensive confounder adjustment. This study aimed to investigate the association between prenatal exposure to ambient PM_2.5_ and lung function at age 8-9 years, identifying critical windows of exposure during pregnancy defined by periods of fetal lung development. Finally, we evaluated effect modification by child sex and allergic sensitization.

## 2. Methods

### 2.1. Study population

This study population included children from the Conditions Affecting Neurocognitive Development and Learning in Early Childhood (CANDLE) cohort, which is one of the prospective cohorts in the ECHO prenatal and early childhood pathways to health consortium (ECHO-PATHWAYS) [21]. CANDLE is a pregnancy cohort, established to identify early life risk factors affecting child neurodevelopment, with participants recruited from 2006 to 2011 [22]. Inclusion criteria for pregnant persons were: age 16–40 years, residence and planned delivery at an affiliated clinic in Shelby County, low-risk singleton pregnancy, and English language proficiency at enrollment. Children were followed in early and middle childhood, including with airway health questionnaires administered to caregivers at age 4–6 and 8–9 years; pulmonary function was measured at age 8–9 years due to the feasibility of spirometry measurement at this age relative to early childhood [23]. Parents provided written informed consent, and children assented to study procedures conducted at age 8–9 years. We included participants with (1) valid spirometry measurements (conducted at the 8–9 visit) and (2) biweekly PM_2.5_ estimates during pregnancy, as described in detail below. We excluded children with gestational age <32 weeks due to the higher rates of bronchopulmonary dysplasia and chronic lung disease in children born very premature [24–26].

The CANDLE study was approved by the University of Tennessee Health Science Center’s Institutional Review Board (IRB) (No. 16-04963-FB). The current analysis was conducted by the ECHO PATHWAYS Consortium and approved by the University of Washington IRB (No. STUDY00000638), in adherence with the Declaration of Helsinki.

### 2.2. Exposures

The primary exposure of interest was prenatal ambient PM_2.5_, derived from a spatiotemporal model yielding average exposure during two-week periods during pregnancy based on longitudinal residential history [27,28]. For the primary analysis, we averaged exposure over gestational weeks representing lung development stages: pseudoglandular (5–16 weeks), canalicular (16–24 weeks), and saccular (24–35 weeks) [11,12]. The alveolar period of development (>35 weeks) was omitted because of the differential length of the averaging period with different gestational ages at delivery. Associations with lung function were scaled to a 2-µg/m^3^ difference in ambient PM_2.5_. We also flexibly explored exposure over continuous 2-week periods during pregnancy in the secondary analyses described below.

### 2.3. Outcomes

Spirometry testing of pulmonary function was performed at the age 8–9 year visit using Breezesuite^TM^ software and based on the American Thoracic Society (ATS) guidelines [29]. Study staff administered a calibrated spirometer for a minimum of 3 and a maximum of 8 attempts for each child. Participant tests were rescheduled if they reported respiratory infections, difficulty breathing, use of asthma/wheeze rescue medication in the three days prior to the exam, or more than one nighttime asthma attack in the past 4 weeks. Post-bronchodilation spirometry was conducted with caregiver consent for children without cardiovascular disorders by administering albuterol and repeating spirometry measurements after 10 min. Lung function results were reviewed by a pulmonologist for quality control, and those deemed unacceptable were removed from the dataset (*N* = 59).

Our primary outcomes of interest were pre-bronchodilation Forced Expiratory Volume in 1 s (FEV1; the volume of air expired in the first second of the blow), pre-bronchodilation Forced Vital Capacity (FVC; the total volume of air that can be forcibly exhaled in one breath after a maximal inspiration), and the pre-bronchodilation ratio of FEV1/FVC. FEV1 and FVC lung function z-scores were calculated using race-neutral Global Lung Initiative (GLI) ‘Global’ reference equations [30] (‘GLI-Global’). In sensitivity analyses, we used 2012 GLI reference equations [31] (‘GLI-2012’) that incorporate child race/ethnicity into z-score estimates, which have been widely used in prior literature. We also conducted sensitivity analyses using raw pulmonary function test values for FEV1 (L), FVC (L), and FEV1/FVC (%). The ­secondary outcome of interest was pre-bronchodilation Forced Expiratory Flow between 25% and 75% of vital capacity (FEF25-75) using GLI-2012 *z*-scores. Post-bronchodilation measurements of FEV1, FVC, and FEV1/FVC were available for 78% of the study population and were considered as outcomes in sensitivity analyses using GLI-Global z-scores. These measures can be used to identify fixed airway obstruction that does not improve with the use of a bronchodilator.

### 2.4. Statistical approach

Our primary statistical approach used linear regression models to evaluate associations between PM_2.5_ exposure averaged across different stages of lung development (pseudoglandular, canalicular, and saccular) and lung function outcomes. Due to potential confounding introduced by the observed correlation between PM_2.5_ concentrations in different exposure windows, our models were mutually adjusted for all three exposure windows [32]. Effect estimates were rescaled to reflect changes associated with a 2-µg/m^3^ unit incremental increase in PM_2.5_, which approximates the interquartile ranges (IQR) and standard deviation (SD) of the exposure distribution in each exposure window. As a secondary analysis, we used Bayesian Distributed Lag Interaction Models [33] (BDLIMs) to flexibly identify associations between PM_2.5_ averaged at 2-week intervals across pregnancy and lung function, as well as potential heterogeneity in associations by pre-specified effect modifiers. BDLIMs were developed to investigate heterogeneity in lagged treatment effects and have the advantage of investigating whether subgroups have the same or different sensitive windows, as well as the magnitude of the effect size in subgroup-specific windows. BDLIM models were implemented using the ‘regimes’ [34] package in R with 80,000 MCMC iterations, using fast covariance estimation (FACE) for smoothing of the covariance matrix, with knots selected to minimize the deviance information criterion (DIC) and default hyperparameters.

Covariates were selected *a priori* and were identified as confounders directly or indirectly associated with prenatal PM_2.5_, child lung function, or as precision variables that were associated with airway health alone and may improve model estimates. Minimally adjusted models included age, sex, and maternal recruitment site (community clinic or hospital). Primary fully adjusted models additionally included maternal education at enrollment (less than high school, high school completion, graduated college/technical school, or any graduate school), neighborhood deprivation index (NDI) averaged across pregnancy, child race (White, Black, or other), child height, household income (continuous; at enrollment adjusted for household size), maternal report of smoking during pregnancy (binary), postnatal environmental smoke exposure (binary), report of recent asthma medication use (binary), and maternal history of asthma (binary). The correlation between PM_2.5_ across birth seasons was variable throughout the study period and was not adequately controlled by birth season and year indicators. Therefore, we used natural splines for the date of birth with four degrees of freedom per year to adjust for seasonality. Extended models were additionally adjusted for potential mediators and additional precision variables: gestational age, birth weight, maternal BMI, firstborn status (binary), the season of outcome assessment, and the ambient concentration of PM_2.5_ in the two weeks prior to spirometry (determined from the model previously described).

Effect modifiers of interest included child sex and allergic sensitization, defined as aeroallergen or food allergen IgE levels >0.35 kU/L in blood samples collected at age 8-9 years. IgE levels were determined by ImmunoCAP-250 using a multiallergen screen for aeroallergens (Phadiatop, mix proprietary) and a food allergen mix (chicken egg, cow’s milk, peanut, soybean, codfish, and wheat) [35–38]. We investigated effect modification with multiplicative interaction terms between PM_2.5_ concentrations in each exposure window and the potential modifier in the mutually adjusted linear regressions. In addition, we investigated interactions in the secondary BDLIM analyses, which can identify potential differences in sensitive windows of exposure between subgroups, as well as the subgroup-specific effect sizes [33]. We ran BDLIM models allowing the overall PM_2.5_ coefficient, time-period-specific PM_2.5_ coefficients, neither, or both to vary across effect modifier groups, choosing the model with the highest mean log posterior predictive density.

In sensitivity analyses, we fit separate linear regression models for each exposure window in association with primary outcomes (rather than models mutually adjusted for all exposure windows) and modeled birth season and birth year using indicator variables and the interaction between them as an alternative to natural splines for calendar time. We also repeated primary analyses after restricting the analytic sample to children without current asthma at the age of 8-9 years, and separately for children with gestational age at birth of at least 37 weeks. We defined current asthma as a positive response to two of three questions on the International Study of Asthma and Allergies in Childhood (ISAAC) Questionnaire: current wheeze (*Has your child ever had wheezing or whistling in the chest in the last 12 months?*), ever physician-diagnosed asthma (*Have you ever been told by a doctor or other health care provider that [child’s name] has asthma or ‘reactive airway disease’?*), and current asthma medication use (*In the past 12 months has your child used any type of medicines, liquids, puffers or other medication for wheezing or asthma?*). Finally, additional sensitivity analyses included pregnancy NO_2_ exposure and natural log-transformed maternal urinary cotinine levels as covariates. Average ambient NO_2_ concentration during pregnancy was determined using the spatiotemporal model described above [27,28]. Cotinine was measured in a spot urine sample collected during mid-pregnancy *via* solid-phase extraction followed by LC-MS/MS.

## 3. Results

### 3.1. Study population characteristics

There were 1503 birth parent-child dyads in the CANDLE cohort, of which 739 completed valid spirometry measurements at the age 8–9 year visit. We excluded five participants with invalid residential histories during pregnancy, nine children with gestational age at birth less than 32 weeks, and 50 children without necessary covariates, for a study sample of 675 dyads. Relative to CANDLE participants who were lost to follow-up or excluded from this analysis, birth parents included in the study population were more likely to be recruited at the hospital (relative to the community clinic), and more likely to have higher educational attainment at enrollment (Table S1).

Children in the study population were approximately 50% male and had a mean age of 8.9 (SD:0.7) years at outcome assessment; 65% were Black or African American, and 28% were white based on parent report (Table 1). Approximately 11% of children were reported to have current asthma at the time of spirometry. Among birth parents, 56% completed high school at the time of enrollment and 17% had a history of asthma. The median adjusted household income at the time of enrollment was $15,000.

**Table 1.** Characteristics of the study population.

	Overall (*N* = 675)
**Child sex**	
Female	344 (51.0%)
Male	331 (49.0%)
**Child age at spirometry (y)**	
Mean (SD)	8.86 (0.736)
Median [Min, Max]	8.80 [8.00, 11.0]
**Child height at spirometry (cm)**	
Mean (SD)	135 (7.76)
Median [Min, Max]	134 [101, 163]
**Recruitment site**	
Community clinic	130 (19.3%)
Hospital	545 (80.7%)
**Child race**	
Black	436 (64.6%)
White	193 (28.6%)
Other	46 (6.8%)
**Current asthma medication use**	
Yes	86 (12.7%)
No	589 (87.3%)
**Current asthma**	
Yes	73 (10.8%)
No	601 (89.0%)
Missing	1 (0.1%)
**Preterm birth**	
Yes	57 (8.4%)
No	618 (91.6%)
**Postnatal smoke exposure**	
Yes	264 (39.1%)
No	411 (60.9%)
**Maternal prenatal smoking**	
Yes	61 (9.0%)
No	614 (91.0%)
**Maternal education at enrollment**	
Less than high school	68 (10.1%)
High school completion	311 (46.1%)
Graduated college or technical school	220 (32.6%)
Some or more graduate school	76 (11.3%)
**Maternal history of asthma**	
Yes	114 (16.9%)
No	561 (83.1%)
**Adjusted household income (USD)**	
Mean (SD)	17700 (13700)
Median [Min, Max]	15000 [626, 100000]
**Neighborhood Deprivation Index**	
Mean (SD)	0.372 (0.885)
Median [Min, Max]	0.372 [-1.22, 3.07]
**Food or aeroallergen IgE >0.35 kU/L**	
Yes	256 (37.9%)
No	202 (29.9%)
Missing	217 (32.1%)

### 3.2. Prenatal PM_2.5_ exposure

Mean ambient PM_2.5_ concentrations during pregnancy ranged from 8.8 µg/m^3^ to 14.6 µg/m^3^, with a mean of 11.1 (SD:1.0) µg/m^3^ (Table 2). We observed notable differences in participants according to exposure to PM_2.5_. Among those in the lowest tertile of pregnancy PM_2.5_ exposure, <1% were recruited from the community clinic compared to 42% in the highest tertile. Participants in the lowest tertile of pregnancy PM_2.5_ exposure were also predominantly recruited later in the study period (99% recruited in 2009 or later compared to 44% in the highest tertile), included more white participants (35% compared to 20% in the highest tertile), and had higher incomes (median $21,700 compared to $13,500 in the highest tertile) (Table S2). The correlation between PM_2.5_ concentrations in different exposure windows ranged from −0.16 to 0.38 (Table S3). There was a low correlation between PM_2.5_ concentrations in different exposure windows and pregnancy average NO_2_ (0.07–0.20) and maternal urinary cotinine (0–0.09).

**Table 2.** Distribution of primary and secondary exposures in the study population.

	Mean	SD	Min	25th Percentile	Median	75th Percentile	Max
PM_2.5_ (µg/m^3^)							
5–16 weeks	10.9	1.9	7.5	9.7	10.4	11.4	18.0
16–24 weeks	11.1	1.9	7.4	9.8	10.6	12.0	18.0
24–35 weeks	11.3	1.9	7.5	10.0	10.9	12.2	17.4
Entire pregnancy	11.1	1.0	8.8	10.2	11.1	11.7	14.6
Secondary exposures							
NO_2_ (ppm)	8.5	2.7	2.5	6.7	8.4	10.2	18.1
Cotinine (log ng/mL)	−0.9	3.4	−6.6	−3.0	−1.2	1.2	7.7

### 3.3. Spirometry outcomes

The mean FEV1 in the study population was 1.66 (SD:0.3) liters (L) (Table 3). Using race-neutral GLI-Global reference equations, the mean FEV1 z-score was determined to be −0.42 (SD:1.05). Using the GLI-2012 reference equations (incorporating child race/ethnicity), the mean FEV1 z-score was 0.30 (SD:1.01). The average FEV1/FVC ratio was 87%. (SD: 6.8%).

**Table 3.** Distribution of spirometry measurements in the study population (*N* = 675).

Outcome	Measurement	Mean	SD	Min	25th Percentile	Median	75th Percentile	Max
*Pre-bronchodilation*								
FEV1	L	1.66	0.30	0.78	1.45	1.64	1.85	2.72
	z-score (GLI-Global)	−0.42	1.05	−3.32	−1.13	−0.45	0.24	3.07
	z-score (GLI-2012)	0.30	1.01	−2.84	−0.34	0.31	0.96	3.25
FVC	L	1.91	0.36	1.07	1.64	1.89	2.12	3.11
	z-score (GLI-Global)	−0.36	1.04	−3.36	−1.12	−0.40	0.34	2.72
	z-score (GLI-2012)	0.40	0.99	−2.57	−0.28	0.43	1.05	3.45
FEV1/FVC	%	87	6.8	60	83	88	92	100
	z-score (GLI-Global)	−0.08	1.12	−3.24	−0.82	−0.08	0.60	2.83
	z-score (GLI-2012)	−0.16	1.09	−3.30	−0.91	−0.20	0.49	2.68
FEF25-75	L/second	1.93	0.54	0.52	1.55	1.89	2.28	3.85
	z-score (GLI-2012)	−0.15	0.95	−3.23	−0.78	−0.17	0.44	3.03
*Post-bronchodilation*								
FEV1	z-score (GLI-Global)	−0.17	1.13	−5.16	−0.91	−0.18	0.52	3.49
FVC	z-score (GLI-Global)	−0.24	1.07	−3.50	−0.96	−0.30	0.49	3.30
FEV1/FVC	z-score (GLI-Global)	0.19	1.16	−3.68	−0.50	0.21	0.91	2.86

L: Liter; FEV1: Forced Expiratory Volume in 1 s; FVC: Forced Vital Capacity; FEF25-75: Forced Expiratory Flow between 25% and 75% of vital capacity.

### 3.4. Analytic results

**Figure 1.**
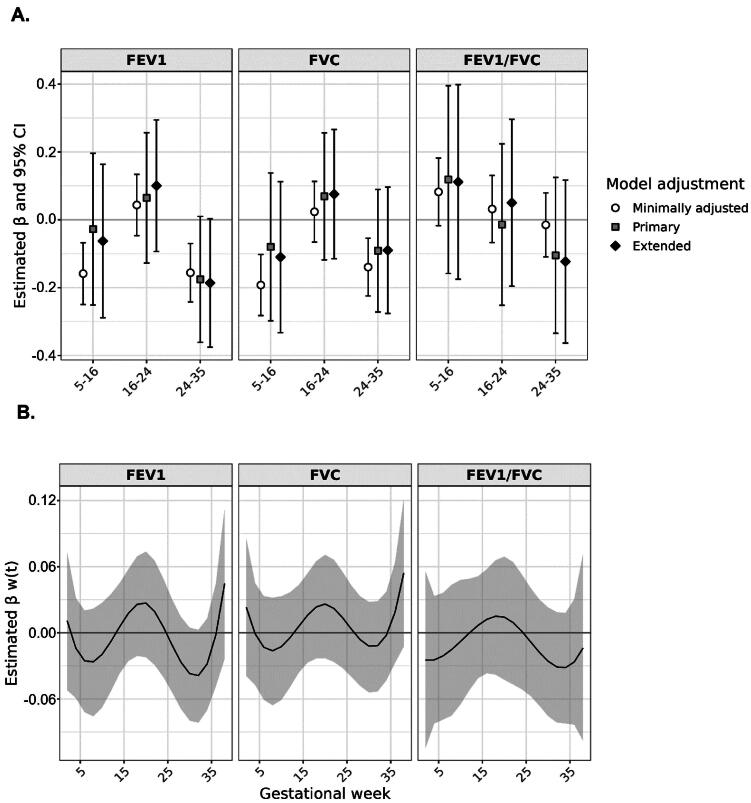
(A) Association between prenatal PM_2.5_ exposure during mutually-adjusted periods of fetal lung development and child lung function. Estimated coefficients (β) and 95% confidence intervals (CI) represent the change in lung function GLI-Global z-scores associated with a 2-µg/m^3^ increase in PM_2.5_ concentration in linear regression models. Minimally adjusted models were adjusted for child age at assessment and child sex. Primary models were additionally adjusted for recruitment site, maternal education at enrollment, NDI, child race, child height, household income, maternal report of smoking during pregnancy, postnatal smoke exposure, recent asthma medication use, and maternal history of asthma. Extended models were additionally adjusted for gestational age, birth weight, maternal BMI, firstborn status, season of outcome assessment, and the ambient concentration of PM_2.5_ in the two weeks prior to spirometry. (B) Results of BDLIM analysis of association between prenatal PM_2.5_ exposure throughout gestation (per 2-µg/m^3^ increase in PM_2.5_ during a given two-week period) and child lung function, using primary model adjustment.

In minimally adjusted models, there was a statistically significant adverse association between prenatal exposure to PM_2.5_ in the pseudoglandular and saccular phases of lung development and the outcome of FEV1, and an adverse association between pseudoglandular exposure and FVC (Figure 1A). Associations were attenuated with primary and extended model adjustments and did not reach statistical significance. However, there was an association approaching significance between prenatal PM_2.5_ exposure in the saccular phase of lung development and FEV1, representing a decrease of 0.176 in FEV1 GLI-Global z-scores associated with a 2 µg/m^3^ increase in mean ambient PM_2.5_, with primary model adjustment (95% CI: −0.361, 0.010). Associations between exposure in the canalicular period of lung development (weeks 16–24) and the outcomes of FEV1 and FVC were generally in the positive direction but close to null and not statistically significant for all models. Associations between prenatal PM_2.5_ exposure and FEV1/FVC were also null under all model specifications. Similar trends were observed in BDLIMs, which flexibly evaluated associations across gestational age. BDLIMs identified adverse associations between PM_2.5_ exposure in early and late pregnancy and the outcome of FEV1; the strongest association was with exposure in week 32 of pregnancy (Figure 1B).

Associations between PM_2.5_ exposure windows and primary lung function outcomes were similar in single-period models, relative to models mutually adjusted for all time periods, although associations between saccular phase PM_2.5_ exposure and FEV1 were statistically significant (−0.191, 95% CI: −0.371, −0.011) (Table S4). Associations were largely unchanged with the use of GLI-2012 z-scores (instead of GLI-Global *z*-scores), alternative specification of time trends with birth year and birth season interaction terms, restriction of the study sample to children without asthma or children born at least 37 weeks of gestation, or adjustment for maternal urinary cotinine levels (Table S4). Following adjustment for maternal prenatal NO_2_ exposure, the adverse associations between PM_2.5_ exposure in the pseudoglandular and saccular periods and the outcomes of FEV1 and FVC were strengthened and mid-pregnancy associations in the positive direction were attenuated (Table S4); with adjustment for NO_2_, the association between saccular phase exposure and FEV1 became statistically significant (−0.217, 95% CI: −0.415, −0.019), though with confidence intervals that overlapped with primary estimates. There was also a borderline significant association between saccular phase PM_2.5_ exposure and raw FEV1 values (−0.044 L per 2-µg/m^3^ increase in PM_2.5_, 95% CI: −0.086, −0.002).

Associations between prenatal PM_2.5_ exposure and the secondary outcome of FEF25-75 were null in models with primary adjustment, although an adverse association was observed for exposure in the saccular period (−0.158, 95% CI: −0.355, 0.039) (Table S5). The results for post-bronchodilation FEV1, FVC, and FEV1/FVC were similar to those for pre-bronchodilation spirometry measures, with adverse associations between exposure to PM_2.5_ in the saccular period and the outcomes of FEV1 and FVC (Table S5).

**Figure 2.**
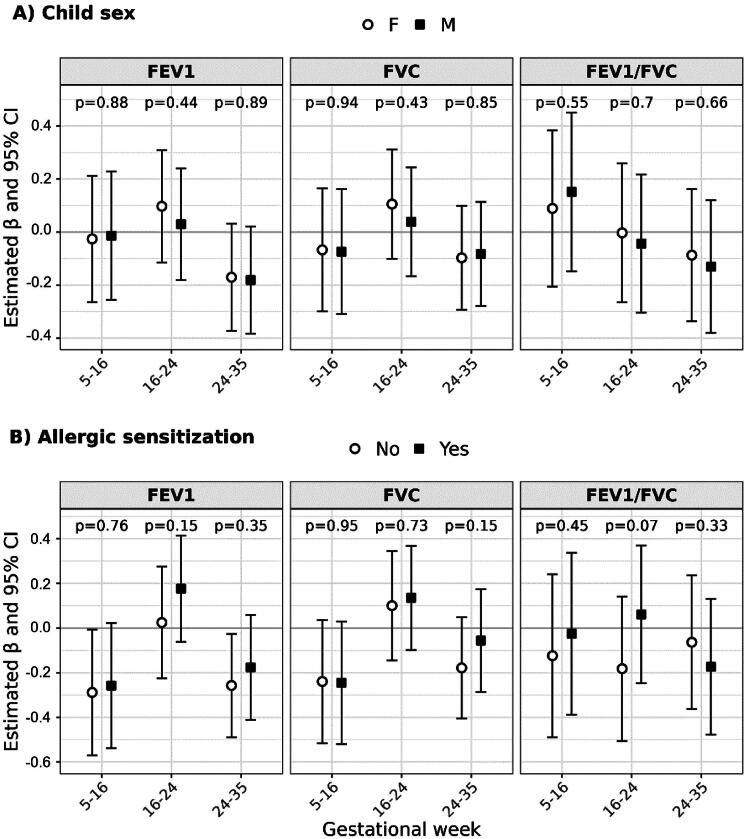
Effect modification by (A) child sex and (B) allergic sensitization in the association between prenatal PM_2.5_ exposure during periods of lung development and child lung function. Estimated coefficients (β) and 95% confidence intervals (CI) show the change in lung function GLI Global z-scores associated with a 2 µg/m^3^ increase in PM_2.5_ concentration. Strata-specific results were derived from interaction models; *p*-values are for the interaction term between the modifier and PM_2.5_ concentrations. Models were adjusted for child age at assessment, child sex, recruitment site, maternal education at enrollment, NDI, child race, child height, household income, maternal report of smoking during pregnancy, postnatal smoke exposure, recent asthma medication use, and maternal history of asthma.

In effect modification analyses, sex-specific differences in exposure-response associations were not observed in linear regression interaction models (Figure 2A) or BDLIM (Figure S1). Associations between PM_2.5_ exposure and the ratio of FEV1/FVC varied between children with and without allergic sensitization, but the direction of association was not consistent and interaction p-values were not statistically significant (Figure 2B, Figure S2).

## 4. Discussion

This study did not reveal strong evidence of associations between prenatal exposure to ambient PM_2.5_ and lung function in children age 8–9 years. However, there were generally adverse associations between PM_2.5_ exposure late pregnancy and the outcomes of FEV1 and FVC. Although not statistically significant in the primary analysis, the association between late pregnancy exposure and FEV1 for exposure during late pregnancy was robust to or strengthened by alternate modeling specifications, suggesting that the saccular phase of lung development may be a vulnerable window for PM_2.5_ exposure. Associations did not appear to be modified by child sex or allergic sensitization.

Our analysis focuses on prenatal PM_2.5_ exposure, which may impact child lung development through indirect pathways including epigenetic changes in gene regulation, DNA methylation, immune function, or maternal systemic inflammation or oxidative stress, which may affect fetal nutrient or oxygen supply [13–15,39]. There is also evidence that particulate matter can cross the placental barrier beginning early in pregnancy, leading to direct impacts on the developing fetus [40]. In animal models, prenatal PM_2.5_ exposure has been found to suppress the expression of genes related to lung development, adversely impacting lung volume and alveolarization [41,42]. Our analysis of prenatal PM_2.5_ exposures identified stronger associations for FEV1 relative to FVC and FEV1/FVC. FEV1 is an indicator of airway obstruction, with lower values corresponding to more severe obstruction [43]. In children with asthma, obstruction is often reversible with the use of bronchodilators. FVC is a proxy for lung volume, with lower values suggestive of restrictive lung disease. A low FEV1/FVC ratio is an indicator of obstruction and airway limitation and diagnostic of asthma severity in children [44]. Stronger associations for FEV1 relative to other measures may suggest that prenatal PM_2.5_ exposure contributes to airway obstruction in middle childhood. These associations were observed in children without asthma and persisted following bronchodilation, pointing towards potential subclinical and non-reversible obstruction following prenatal PM_2.5_ exposure.

Prior studies of prenatal PM_2.5_ exposure and lung function have also generally identified adverse associations with both FEV1 and FVC. However, these studies have used varied methods of exposure assessment, age of outcome assessment, and outcome metrics, limiting comparison to the present study. Higher levels of personal 48-hour PM_2.5_ measurements in the second trimester of pregnancy were significantly associated with lower forced expiratory volume in 1 s (FEV1) and forced vital capacity (FVC) at 4–9 years among a cohort of around 250 children in Poland [6,7]. In a cohort of 222 children from Fresno, CA, an increase of approximately 5 µg/m^3^ PM_2.5_ was associated with 0.42 L lower FVC (95% CI: −0.81, −0.03) and 0.38 L lower FEV1 (95% CI: −0.74, −0.01), with stronger associations in the second half of pregnancy [45]. Among 230 children in a Boston birth cohort, daily PM_2.5_ during late pregnancy was weakly associated with impaired lung function at age 7 in boys [9]. In contrast, Stapleton et al. [10] did not identify adverse associations between prenatal PM_2.5_ exposure and child lung function among approximately 400 children in Sabadell, Spain, although significant associations were identified for coarse PM (PM_10_).

Two of the aforementioned studies identified stronger associations in late pregnancy relative to early pregnancy [9,45], consistent with our findings of a potentially vulnerable window during saccular lung development. Both prior studies used flexible distributed lag models to identify the critical windows of exposure. We used complementary approaches of linear regression with prespecified exposure windows driven by prior knowledge of fetal airway development and the flexible BDLIM model. Both approaches suggested an adverse association in late pregnancy. We also identified adverse associations in early pregnancy, although these associations did not reach statistical significance in any of the model specifications. This dual approach utilizing both linear and distributed lag models is a unique strength of the analysis.

Prior findings regarding potentially vulnerable subpopulations have been mixed. Lee et al. [9] found a stronger association between late-pregnancy exposure to PM_2.5_ and child lung function among boys, while other studies have found no difference [7,18]. Studies of postnatal short-term exposure to PM_2.5_ and lung function have also identified stronger associations among boys; differences have been proposed to reflect behavioral differences that influence exposure (outdoor time, outdoor time with high activity, and high respiratory rate) as well as biological/mechanistic differences in lung developmental toxicity [5].

Prior studies of the relationship between prenatal PM_2.5_ exposure and child lung function have rarely considered the role of co-exposure to other air pollutants. Prenatal exposure to nitrogen oxides has been associated with reduced childhood lung function in multiple studies [18–20,46]. In our analysis, additional adjustment for prenatal NO_2_ exposure, a marker of traffic pollution, strengthened the association between early and late pregnancy PM_2.5_ exposure and the outcomes of FEV1 and FVC, although the confidence intervals overlapped with those of the primary analysis. In contrast, some prior studies of child asthma and other airway outcomes have found that associations with prenatal PM exposure have been attenuated by mutual adjustment for other air pollutants, including NO_2_ [47,48]. Antenatal maternal smoking and prenatal exposure to environmental tobacco smoke have also been consistently linked to impaired lung function [49,50]. Our findings were unchanged after adjusting for maternal urinary cotinine as a proxy for tobacco exposure, though cotinine measures were available only for a single time point in mid-pregnancy.

This study has certain limitations that may affect inferences. Our estimates of PM_2.5_ exposure represent modeled ambient conditions outside the primary residence during pregnancy. Prenatal PM_2.5_ exposure may also correlate with long-term postnatal exposure, which was not accounted for in our statistical analysis and could therefore contribute to the observed associations. Future studies would benefit from additional PM_2.5_ measurements and modeling to more accurately assess total early life exposure to PM_2.5_ and other air pollutants of ambient origin. The outcome assessment in our study also relied on a single spirometry measurement; thus, we were unable to evaluate associations with lung function growth trajectories.

This study also has notable strengths. Our sample size is the largest to date among studies exploring the association between prenatal PM_2.5_ and lung function. We included a demographically diverse population that is majority Black or African American, a group historically underrepresented in similar literature. Furthermore, our study population was well characterized, allowing for comprehensive confounder adjustment. We also leverage a well-validated, point-scale spatiotemporal model of ambient PM_2.5_ exposure [27,28] that builds on prior studies using short-term personal monitoring [6,7], land-use-regression models [10,19], or local monitoring records [45,51]. We also used a race-neutral approach to calculate lung function z-scores, in contrast to prior methods that assume genetic origins of racial disparities in lung function [30]. Finally, we employed two complementary approaches to triangulate the critical windows of exposure: mutually adjusted linear regression models and flexible BDLIM model. Both methods indicated potential vulnerability during the saccular phase of fetal lung development. Future confirmatory studies of associations between early life exposure to PM_2.5_ and lung function in children would strengthen evidence regarding the critical window of exposure during pregnancy and early life.

## Supplementary Material

Supplemental Material

## Data Availability

The data used for this study are not publicly available, but de-identified data may be available on request, subject to approval by the internal review board and under a formal data use agreement. Contact the corresponding author for additional information. The computing code in R can be obtained from the corresponding author *via* email.
