# Supplementary material for: Prenatal exposure to ambient fine particulate matter and child lung function in the CANDLE cohort
Source: Ann Med. 2024 Nov 4;56(1):2422051. doi: 10.1080/07853890.2024.2422051 (PMC11536642; doi:10.1080/07853890.2024.2422051)
Supplement: Supplemental Material [file IANN_A_2422051_SM1795.docx]

**Contents**

Table S1. Characteristics of CANDLE participants by inclusion in study population.

Table S2. Study population characteristics by tertile of mean PM_2.5_ exposure during pregnancy.

Table S3. Correlation between PM2.5 exposure in different exposure periods, PM2.5 and NO2 across pregnancy, and mid-pregnancy cotinine.

Table S4. Associations between prenatal PM_2.5_ and child lung function in primary and sensitivity analyses.

Table S5. Associations between prenatal PM_2.5_ and secondary outcome measures.


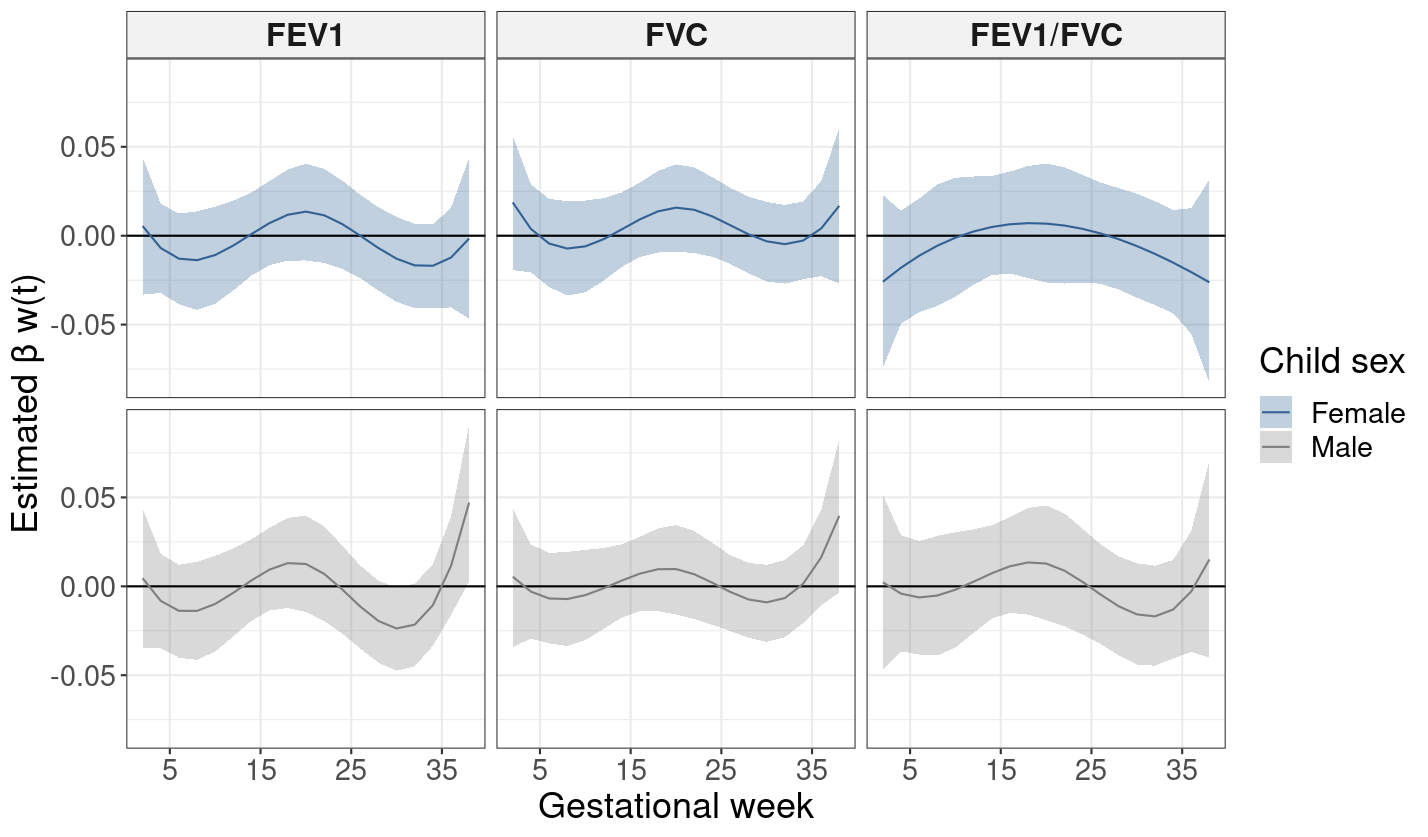
Figure S1. Associations between prenatal PM_2.5_ and child lung function among female and male children in BDLIM models.


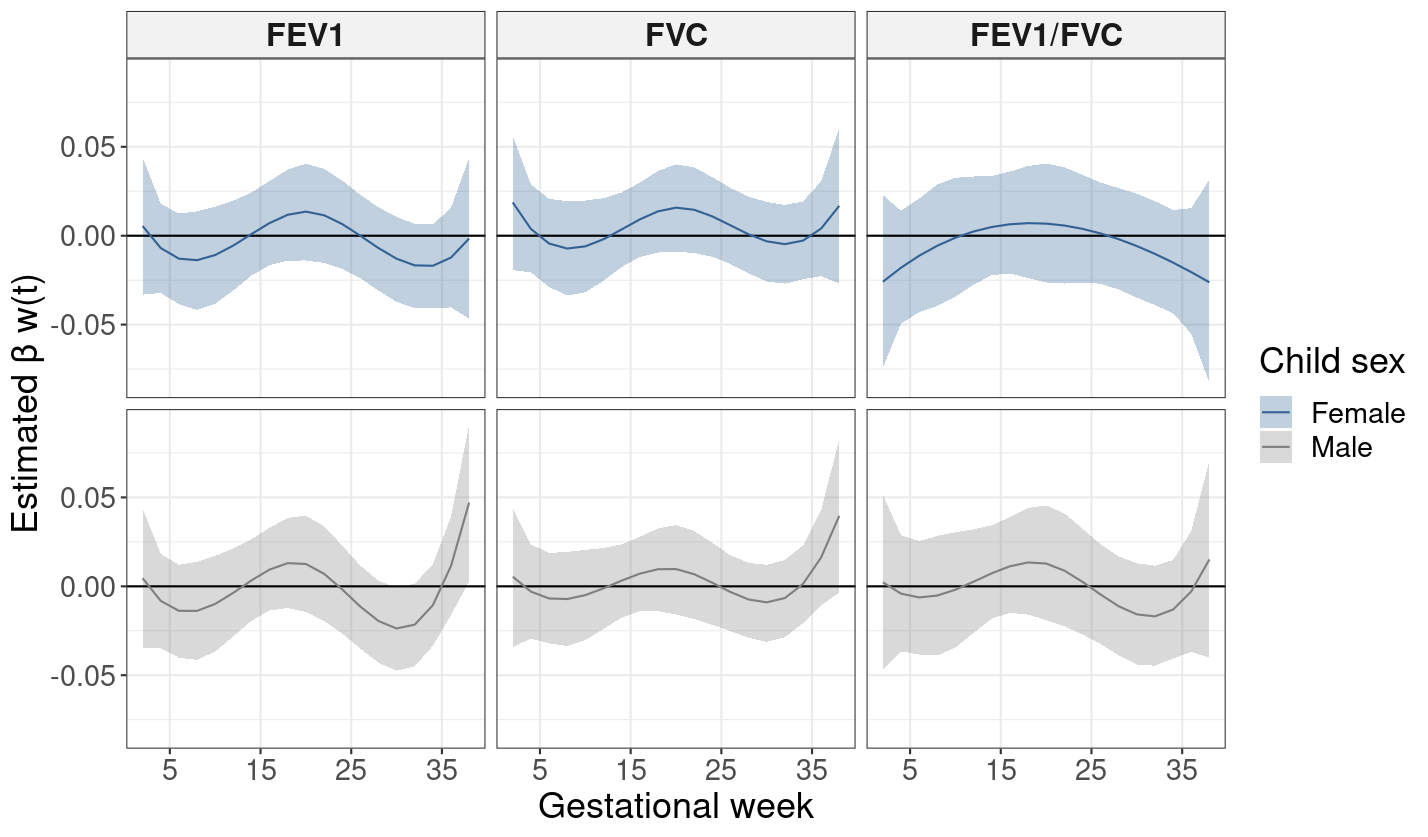
Figure S2. Associations between prenatal PM_2.5_ and child lung function among children with and without allergic sensitization (food or aeroallergen IgE levels >= 0.35 kU/L) in BDLIM models.

Table S1. Characteristics of CANDLE participants by inclusion in study population.

|  | Not included (N = 828) | Included (N = 675) |
| --- | --- | --- |
| **Child sex** |  |  |
| Female | 383 (46.3%) | 344 (51.0%) |
| Male | 405 (48.9%) | 331 (49.0%) |
| Missing | 40 (4.8%) | 0 (0%) |
| **Child age at spirometry (y)** |  |  |
| Mean (SD) | 8.98 (0.869) | 8.86 (0.734) |
| Median [Min, Max] | 8.85 [8.00, 11.0] | 8.80 [8.00, 11.0] |
| Missing | 704 (85.0%) | 0 (0%) |
| **Child height at spirometry (cm)** |  |  |
| Mean (SD) | 136 (7.67) | 135 (7.78) |
| Median [Min, Max] | 135 [120, 154] | 134 [101, 163] |
| Missing | 704 (85.0%) | 0 (0%) |
| **Recruitment site** |  |  |
| Community clinic | 213 (25.7%) | 130 (19.3%) |
| Hospital | 615 (74.3%) | 545 (80.7%) |
| **Child race** |  |  |
| Black | 321 (38.8%) | 435 (64.4%) |
| White | 143 (17.3%) | 193 (28.6%) |
| Other | 25 (3.0%) | 47 (7.0%) |
| Missing | 339 (40.9%) | 0 (0.0%) |
| **Current asthma medication use** |  |  |
| Yes | 41 (5.0%) | 86 (12.7%) |
| No | 195 (23.6%) | 589 (87.3%) |
| Missing | 592 (71.5%) | 0 (0%) |
| **Current asthma** |  |  |
| Yes | 39 (4.7%) | 73 (10.8%) |
| No | 196 (23.7%) | 601 (89.0%) |
| Missing | 593 (71.6%) | 1 (0.1%) |
| **Preterm birth** |  |  |
| Yes | 76 (9.2%) | 57 (8.4%) |
| No | 705 (85.1%) | 618 (91.6%) |
| Missing | 47 (5.7%) | 0 (0%) |
| **Postnatal smoke exposure** |  |  |
| Yes | 182 (22.0%) | 264 (39.1%) |
| No | 151 (18.2%) | 411 (60.9%) |
| Missing | 495 (59.8%) | 0 (0%) |
| **Maternal prenatal smoking** |  |  |
| Yes | 90 (10.9%) | 61 (9.0%) |
| No | 737 (89.0%) | 614 (91.0%) |
| Missing | 1 (0.1%) | 0 (0%) |
| **Maternal education at enrollment** |  |  |
| Less than high school | 117 (14.1%) | 67 (9.9%) |
| High school completion | 398 (48.1%) | 311 (46.1%) |
| Graduated college or technical school | 217 (26.2%) | 220 (32.6%) |
| Some or more graduate school | 95 (11.5%) | 76 (11.3%) |
| Missing | 1 (0.1%) | 1 (0.1%) |
| **Maternal history of asthma** |  |  |
| Yes | 93 (11.2%) | 114 (16.9%) |
| No | 425 (51.3%) | 561 (83.1%) |
| Missing | 310 (37.4%) | 0 (0%) |
| **Adjusted household income (USD)** |  |  |
| Mean (SD) | 18400 (14300) | 17700 (13700) |
| Median [Min, Max] | 15000 [751, 50000] | 15000 [626, 100000] |
|  | 645 (77.9%) | 0 (0%) |
| **Neighborhood Deprivation Index** |  |  |
| Mean (SD) | 0.343 (0.834) | 0.373 (0.886) |
| Median [Min, Max] | 0.309 [-1.04, 3.07] | 0.372 [-1.22, 3.07] |
|  | 75 (9.1%) | 0 (0%) |
| **Food or aeroallergen IgE >0.35 kU/L** |  |  |
| Yes | 48 (5.8%) | 256 (37.9%) |
| No | 39 (4.7%) | 202 (29.9%) |
| Missing | 741 (89.5%) | 217 (32.1%) |

Table S2. Study population characteristics by tertile of mean PM_2.5_ exposure during pregnancy

|  | 1 (N=225) | 2 (N=225) | 3 (N=225) | Overall (N=675) |
| --- | --- | --- | --- | --- |
| **Child sex** |  |  |  |  |
| Female | 114 (50.7%) | 109 (48.4%) | 121 (53.8%) | 344 (51.0%) |
| Male | 111 (49.3%) | 116 (51.6%) | 104 (46.2%) | 331 (49.0%) |
| **Child age at spirometry (y)** |  |  |  |  |
| Mean (SD) | 8.76 (0.592) | 8.60 (0.695) | 9.21 (0.771) | 8.86 (0.736) |
| Median [Min, Max] | 8.70 [8.00, 10.7] | 8.20 [8.00, 11.0] | 9.20 [8.00, 11.0] | 8.80 [8.00, 11.0] |
| **Child height at spirometry (cm)** |  |  |  |  |
| Mean (SD) | 134 (7.60) | 134 (7.54) | 137 (7.74) | 135 (7.76) |
| Median [Min, Max] | 134 [115, 163] | 134 [101, 159] | 136 [114, 158] | 134 [101, 163] |
| **Recruitment site** |  |  |  |  |
| Community clinic | 2 (0.9%) | 32 (14.2%) | 96 (42.7%) | 130 (19.3%) |
| Hospital | 223 (99.1%) | 193 (85.8%) | 129 (57.3%) | 545 (80.7%) |
| **Child race** |  |  |  |  |
| Black | 127 (56.4%) | 138 (61.3%) | 171 (76.0%) | 436 (64.6%) |
| White | 81 (36.0%) | 69 (30.7%) | 43 (19.1%) | 193 (28.6%) |
| Other | 17 (7.6%) | 18 (8.0%) | 11 (4.9%) | 46 (6.8%) |
| **Asthma medication use** |  |  |  |  |
| Yes | 29 (12.9%) | 30 (13.3%) | 27 (12.0%) | 86 (12.7%) |
| No | 196 (87.1%) | 195 (86.7%) | 198 (88.0%) | 589 (87.3%) |
| **Current asthma** |  |  |  |  |
| Yes | 26 (11.6%) | 25 (11.1%) | 22 (9.8%) | 73 (10.8%) |
| No | 199 (88.4%) | 199 (88.4%) | 203 (90.2%) | 601 (89.0%) |
| Missing | 0 (0%) | 1 (0.4%) | 0 (0%) | 1 (0.1%) |
| **Preterm birth** |  |  |  |  |
| Yes | 15 (6.7%) | 17 (7.6%) | 25 (11.1%) | 57 (8.4%) |
| No | 210 (93.3%) | 208 (92.4%) | 200 (88.9%) | 618 (91.6%) |
| **Postnatal ETS exposure** |  |  |  |  |
| Yes | 68 (30.2%) | 93 (41.3%) | 103 (45.8%) | 264 (39.1%) |
| No | 157 (69.8%) | 132 (58.7%) | 122 (54.2%) | 411 (60.9%) |
| **Maternal prenatal smoking** |  |  |  |  |
| Yes | 15 (6.7%) | 20 (8.9%) | 26 (11.6%) | 61 (9.0%) |
| No | 210 (93.3%) | 205 (91.1%) | 199 (88.4%) | 614 (91.0%) |
| **Maternal education at enrollment** |  |  |  |  |
| Less than high school | 10 (4.4%) | 23 (10.2%) | 35 (15.6%) | 68 (10.1%) |
| High school completion | 92 (40.9%) | 103 (45.8%) | 116 (51.6%) | 311 (46.1%) |
| Graduated college or technical school | 92 (40.9%) | 71 (31.6%) | 57 (25.3%) | 220 (32.6%) |
| Some or more graduate school | 31 (13.8%) | 28 (12.4%) | 17 (7.6%) | 76 (11.3%) |
| **Maternal history of asthma** |  |  |  |  |
| Yes | 39 (17.3%) | 37 (16.4%) | 38 (16.9%) | 114 (16.9%) |
| No | 186 (82.7%) | 188 (83.6%) | 187 (83.1%) | 561 (83.1%) |
| **Adjusted household income (USD)** |  |  |  |  |
| Mean (SD) | 21700 (14100) | 18000 (14800) | 13400 (10800) | 17700 (13700) |
| Median [Min, Max] | 17500 [1880, 62500] | 15000 [626, 100000] | 10000 [626, 62500] | 15000 [626, 100000] |
| **Neighborhood Deprivation Index** |  |  |  |  |
| Mean (SD) | 0.155 (0.823) | 0.287 (0.887) | 0.673 (0.864) | 0.372 (0.885) |
| Median [Min, Max] | -0.0361 [-0.963, 2.80] | 0.207 [-1.22, 3.07] | 0.790 [-1.22, 2.80] | 0.372 [-1.22, 3.07] |
| **Birth year** |  |  |  |  |
| 2007 | 0 (0%) | 11 (4.9%) | 38 (16.9%) | 49 (7.3%) |
| 2008 | 2 (0.9%) | 33 (14.7%) | 87 (38.7%) | 122 (18.1%) |
| 2009 | 116 (51.6%) | 17 (7.6%) | 31 (13.8%) | 164 (24.3%) |
| 2010 | 84 (37.3%) | 62 (27.6%) | 31 (13.8%) | 177 (26.2%) |
| 2011 | 23 (10.2%) | 102 (45.3%) | 38 (16.9%) | 163 (24.1%) |
| **Birth season** |  |  |  |  |
| Winter | 29 (12.9%) | 14 (6.2%) | 73 (32.4%) | 116 (17.2%) |
| Spring | 89 (39.6%) | 62 (27.6%) | 24 (10.7%) | 175 (25.9%) |
| Summer | 59 (26.2%) | 94 (41.8%) | 46 (20.4%) | 199 (29.5%) |
| Autumn | 48 (21.3%) | 55 (24.4%) | 82 (36.4%) | 185 (27.4%) |

Table S3. Correlation between PM_2.5_ exposure in different exposure periods, PM_2.5_ and NO_2_ across pregnancy, and mid-pregnancy cotinine

|  | PM_2.5_ (5-16 weeks) | PM_2.5_ (16-24 weeks) | PM_2.5_ (24-35 weeks) | PM_2.5_ (Entire pregnancy) | NO_2_ (Entire pregnancy) | Cotinine |
| --- | --- | --- | --- | --- | --- | --- |
| PM_2.5_ (5-16 weeks) | 1 | 0.38 | -0.16 | 0.66 | 0.20 | 0.09 |
| PM_2.5_ (16-24 weeks) | 0.38 | 1 | 0.23 | 0.62 | 0.07 | 0.00 |
| PM_2.5_ (24-35 weeks) | -0.16 | 0.23 | 1 | 0.54 | 0.20 | -0.02 |
| PM_2.5_ (Entire pregnancy) | 0.66 | 0.62 | 0.54 | 1 | 0.42 | 0.08 |
| NO_2_ (Entire pregnancy)) | 0.20 | 0.07 | 0.20 | 0.42 | 1 | 0.12 |
| Cotinine | 0.09 | 0.00 | -0.02 | 0.08 | 0.12 | 1 |


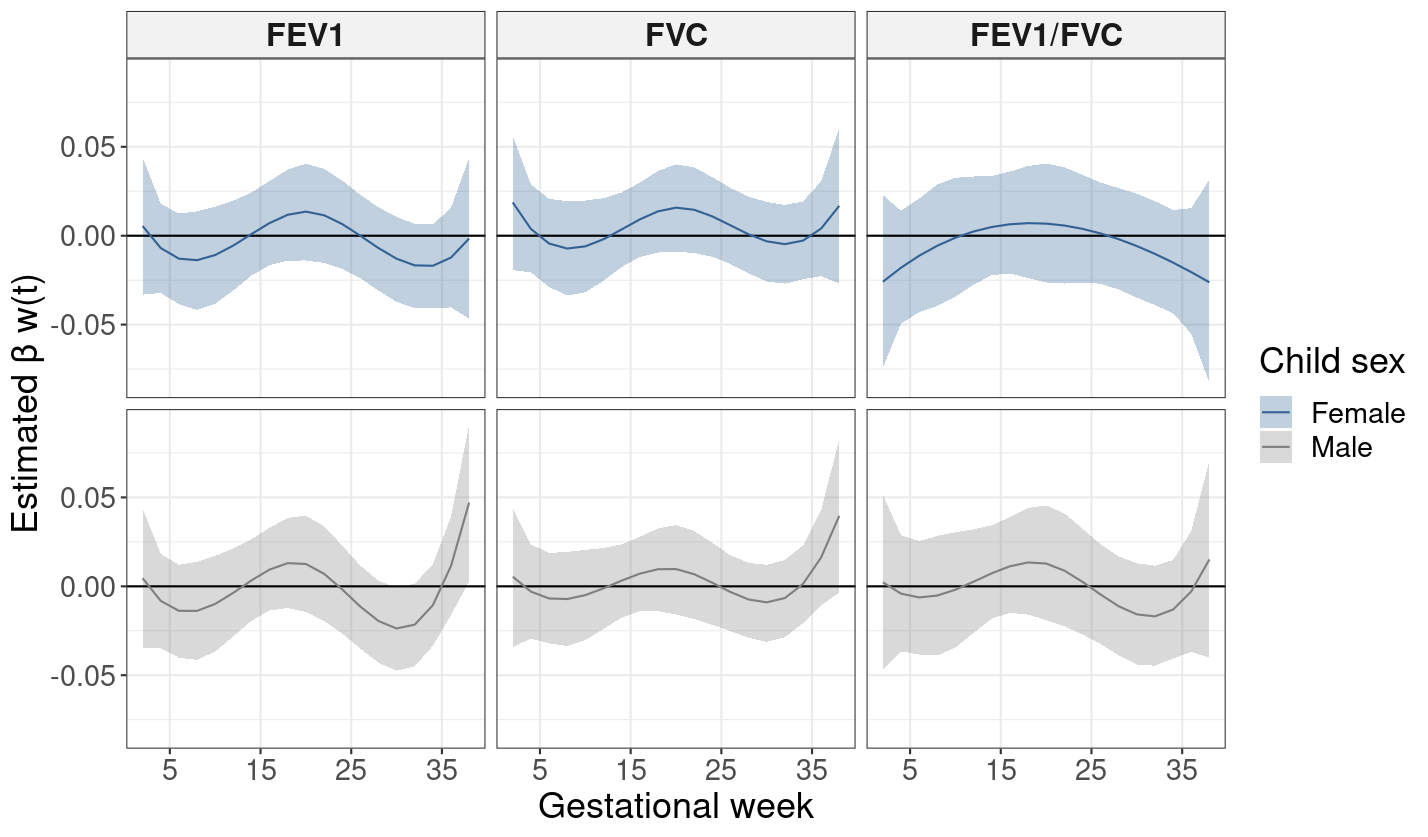


Table S4. Associations between prenatal PM_2.5_ and child lung function in primary and sensitivity analyses. Estimated coefficients (β) and 95% confidence intervals (CI) represent the change in lung function GLI-Global z-scores associated with a 2 µg/m^3^ increase in PM_2.5_ concentration in linear regression models. Models were adjusted for child age at assessment, child sex, recruitment site, maternal education at enrollment, NDI, child race, child height, household income, maternal report of smoking during pregnancy, postnatal smoke exposure, recent asthma medication use, and maternal history of asthma.

|  |  | Exposure window (gestational weeks) | | |
| --- | --- | --- | --- | --- |
| Analysis | Outcome | 5-16 | 16-24 | 24-35 |
| Primary models | FEV1 | -0.049 (-0.26, 0.162) | 0.057 (-0.124, 0.239) | -0.183 (-0.361, -0.005) |
|  | FVC | -0.109 (-0.315, 0.098) | 0.069 (-0.109, 0.246) | -0.089 (-0.263, 0.085) |
|  | FEV1/FVC | 0.118 (-0.147, 0.384) | -0.029 (-0.257, 0.199) | -0.123 (-0.347, 0.101) |
| Single exposure period models | FEV1 | -0.042 (-0.264, 0.180) | 0.108 (-0.077, 0.293) | -0.191 (-0.37, -0.011) |
|  | FVC | -0.093 (-0.309, 0.123) | 0.100 (-0.081, 0.280) | -0.107 (-0.283, 0.068) |
|  | FEV1/FVC | 0.117 (-0.157, 0.391) | -0.006 (-0.235, 0.223) | -0.101 (-0.324, 0.122) |
| GLI-2012 z-scores | FEV1 | -0.056 (-0.306, 0.194) | 0.064 (-0.151, 0.279) | -0.192 (-0.400, 0.015) |
|  | FVC | -0.109 (-0.356, 0.137) | 0.065 (-0.148, 0.277) | -0.099 (-0.303, 0.106) |
|  | FEV1/FVC | 0.120 (-0.150, 0.391) | 0.003 (-0.230, 0.235) | -0.092 (-0.316, 0.133) |
| Seasonal adjustment: Birth year*Birth season | FEV1 | -0.128 (-0.288, 0.033) | 0.098 (-0.035, 0.231) | -0.111 (-0.243, 0.021) |
|  | FVC | -0.147 (-0.304, 0.009) | 0.055 (-0.074, 0.185) | -0.099 (-0.228, 0.029) |
|  | FEV1/FVC | 0.057 (-0.142, 0.255) | 0.066 (-0.099, 0.230) | 0.006 (-0.157, 0.168) |
| Children without asthma | FEV1 | -0.018 (-0.257, 0.221) | 0.052 (-0.154, 0.259) | -0.155 (-0.354, 0.043) |
|  | FVC | -0.073 (-0.305, 0.159) | 0.090 (-0.110, 0.290) | -0.071 (-0.264, 0.122) |
|  | FEV1/FVC | 0.103 (-0.187, 0.394) | -0.116 (-0.367, 0.134) | -0.126 (-0.367, 0.115) |
| Children born >= 37 weeks gestation | FEV1 | 0.038 (-0.213, 0.288) | 0.120 (-0.089, 0.330) | -0.115 (-0.322, 0.092) |
|  | FVC | -0.043 (-0.285, 0.199) | 0.142 (-0.061, 0.344) | -0.030 (-0.230, 0.170) |
|  | FEV1/FVC | 0.156 (-0.158, 0.469) | -0.034 (-0.297, 0.228) | -0.086 (-0.345, 0.173) |
| Additional adjustment for pregnancy NO_2_ exposure | FEV1 | -0.089 (-0.332, 0.154) | 0.012 (-0.196, 0.220) | -0.219 (-0.417, -0.021) |
|  | FVC | -0.157 (-0.393, 0.079) | 0.004 (-0.198, 0.206) | -0.147 (-0.339, 0.046) |
|  | FEV1/FVC | 0.153 (-0.147, 0.454) | 0.014 (-0.244, 0.272) | -0.077 (-0.323, 0.168) |
| Additional adjustment for pregnancy ETS exposure (urinary cotinine) | FEV1 | -0.023 (-0.251, 0.204) | 0.079 (-0.116, 0.274) | -0.179 (-0.369, 0.011) |
|  | FVC | -0.074 (-0.295, 0.148) | 0.076 (-0.114, 0.267) | -0.089 (-0.274, 0.096) |
|  | FEV1/FVC | 0.115 (-0.168, 0.397) | -0.002 (-0.244, 0.240) | -0.113 (-0.349, 0.122) |

Table S5. Associations between prenatal PM_2.5_ and secondary outcome measures. Estimated coefficients (β) and 95% confidence intervals (CI) represent the change in lung function GLI-Global z-scores associated with a 2 µg/m^3^ increase in PM_2.5_ concentration in linear regression models. Models were adjusted for child age at assessment, child sex, recruitment site, maternal education at enrollment, NDI, child race, child height, household income, maternal report of smoking during pregnancy, postnatal smoke exposure, recent asthma medication use, and maternal history of asthma.

|  |  | Exposure window (gestational weeks) | | |
| --- | --- | --- | --- | --- |
| Outcome |  | 5-16 | 16-24 | 24-35 |
| Pre-bronchodilation | FEF25-75 | 0.054 (-0.184, 0.291) | -0.003 (-0.207, 0.201) | -0.159 (-0.355, 0.038) |
| Post-bronchodilation spirometry measures | FEV1 | -0.084 (-0.374, 0.205) | 0.076 (-0.182, 0.334) | -0.149 (-0.395, 0.096) |
|  | FVC | -0.029 (-0.286, 0.228) | 0.077 (-0.151, 0.305) | -0.176 (-0.393, 0.041) |
|  | FEV1/FVC | -0.021 (-0.344, 0.303) | 0.002 (-0.285, 0.290) | 0.032 (-0.243, 0.306) |


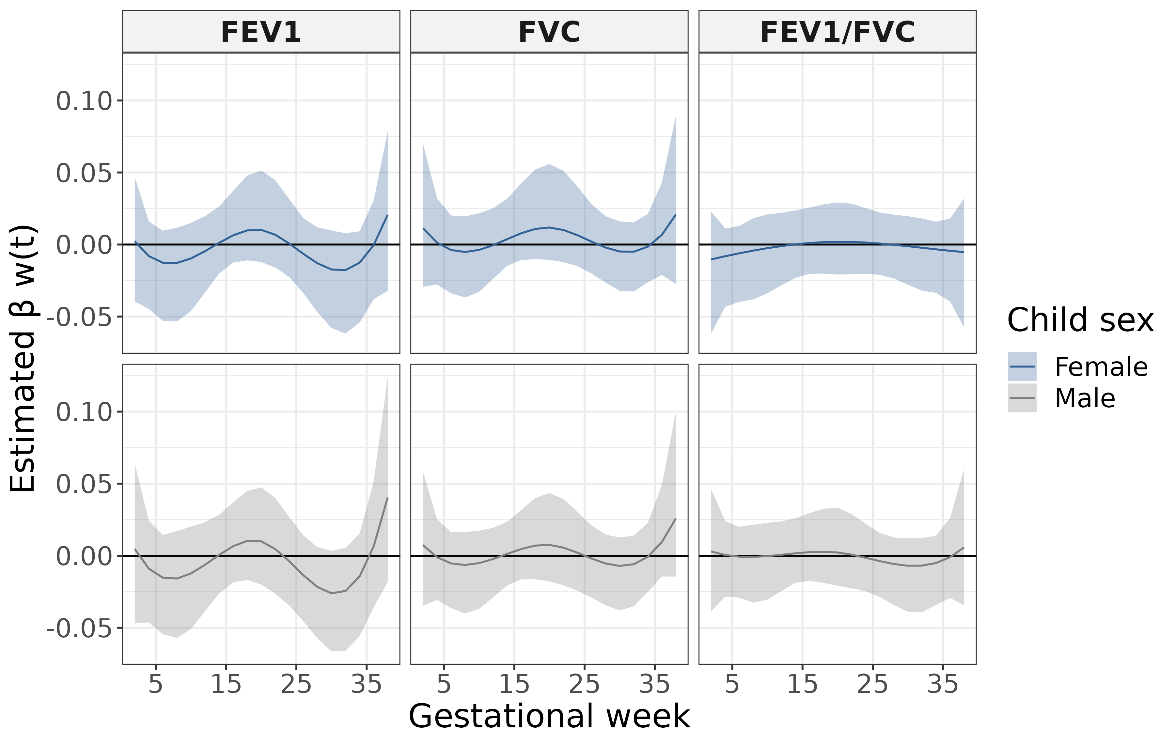


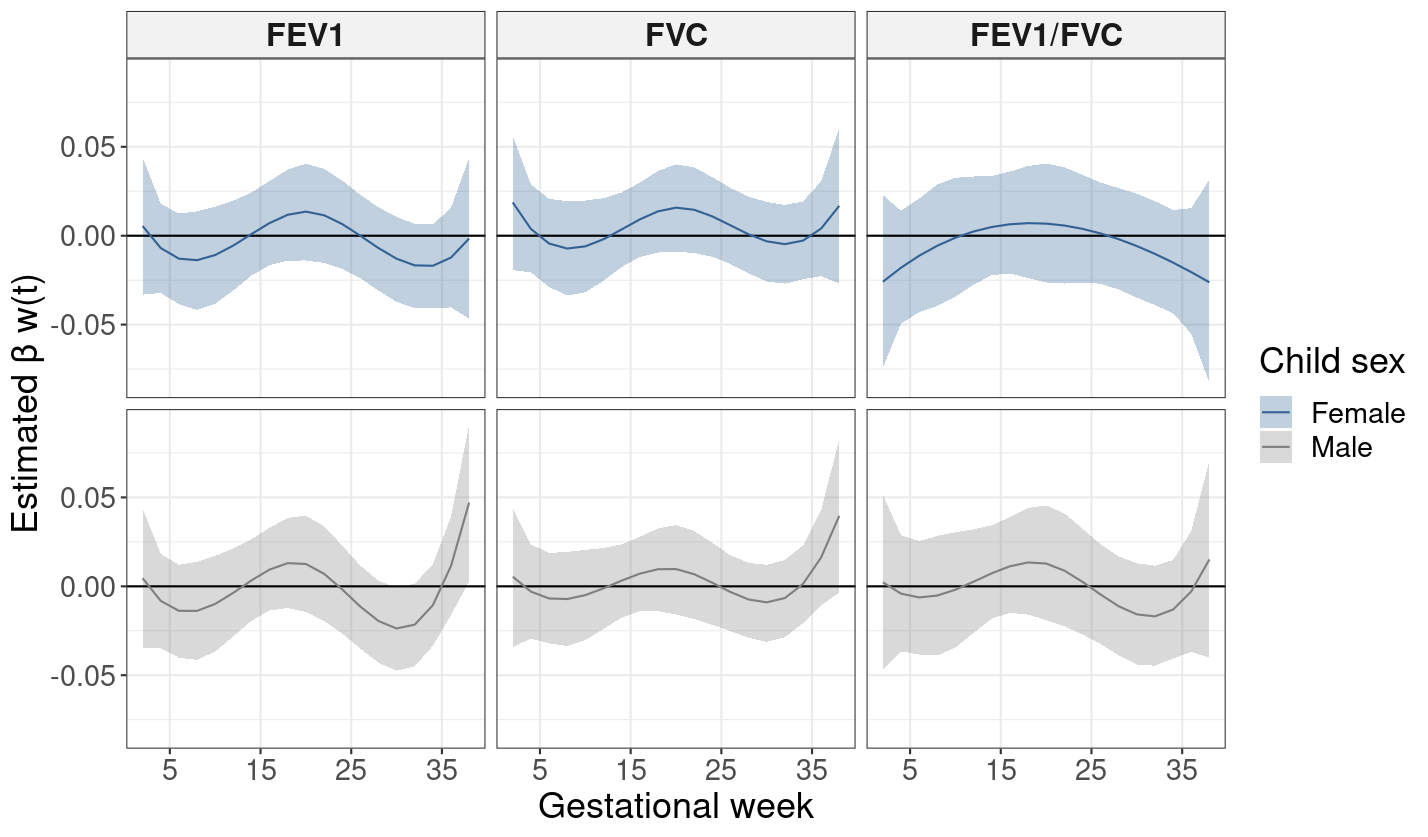
Figure S1. Associations between prenatal PM_2.5_ and child lung function (per 2-µg/m^3^ increase in PM_2.5_ during a given two-week period) among female and male children in BDLIM models. For FEV1 and FVC, the model with the highest posterior predictive density was one in which only the time-varying scalar effect size *β* varied by child sex. For FEV1/FVC, the model with the highest posterior predictive density was one in which only the time-varying weight function *w(t)* varied by child sex.


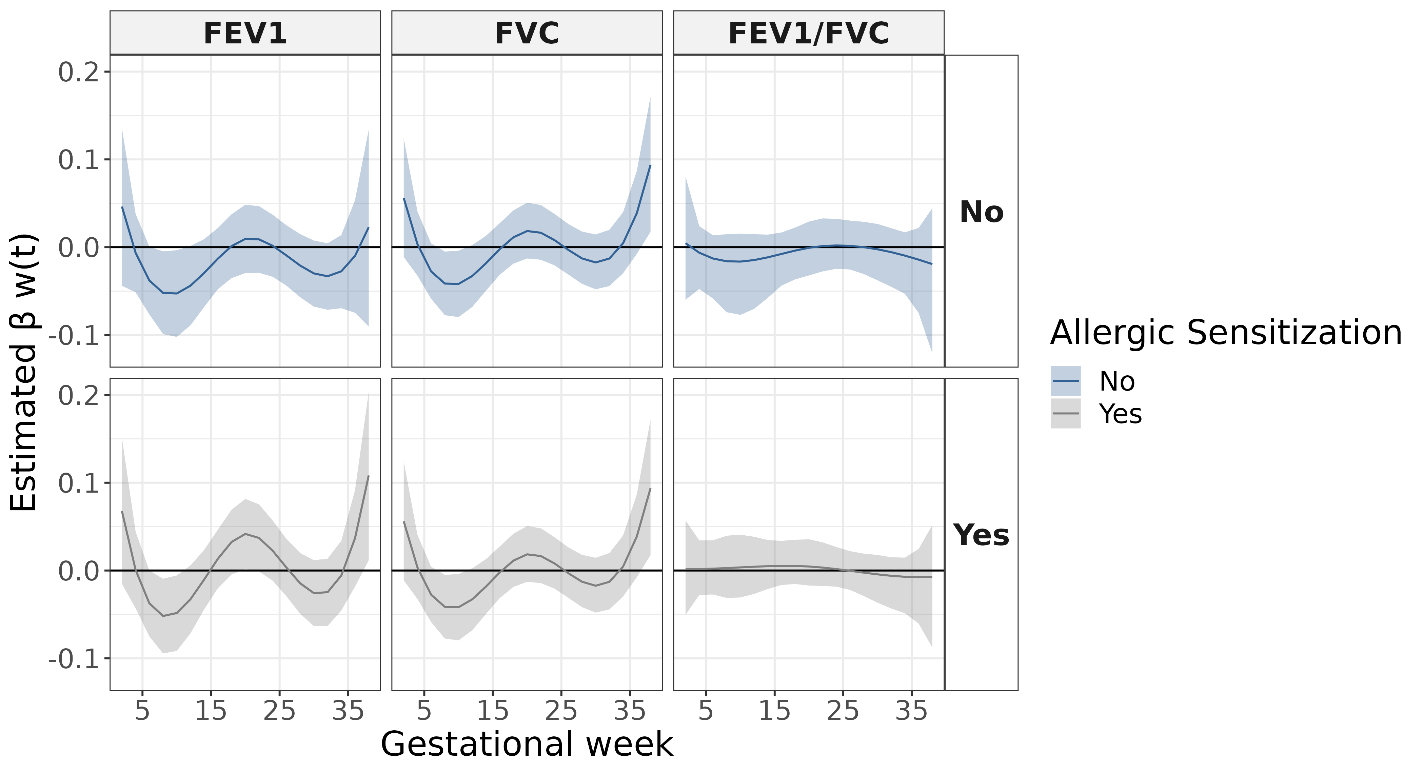


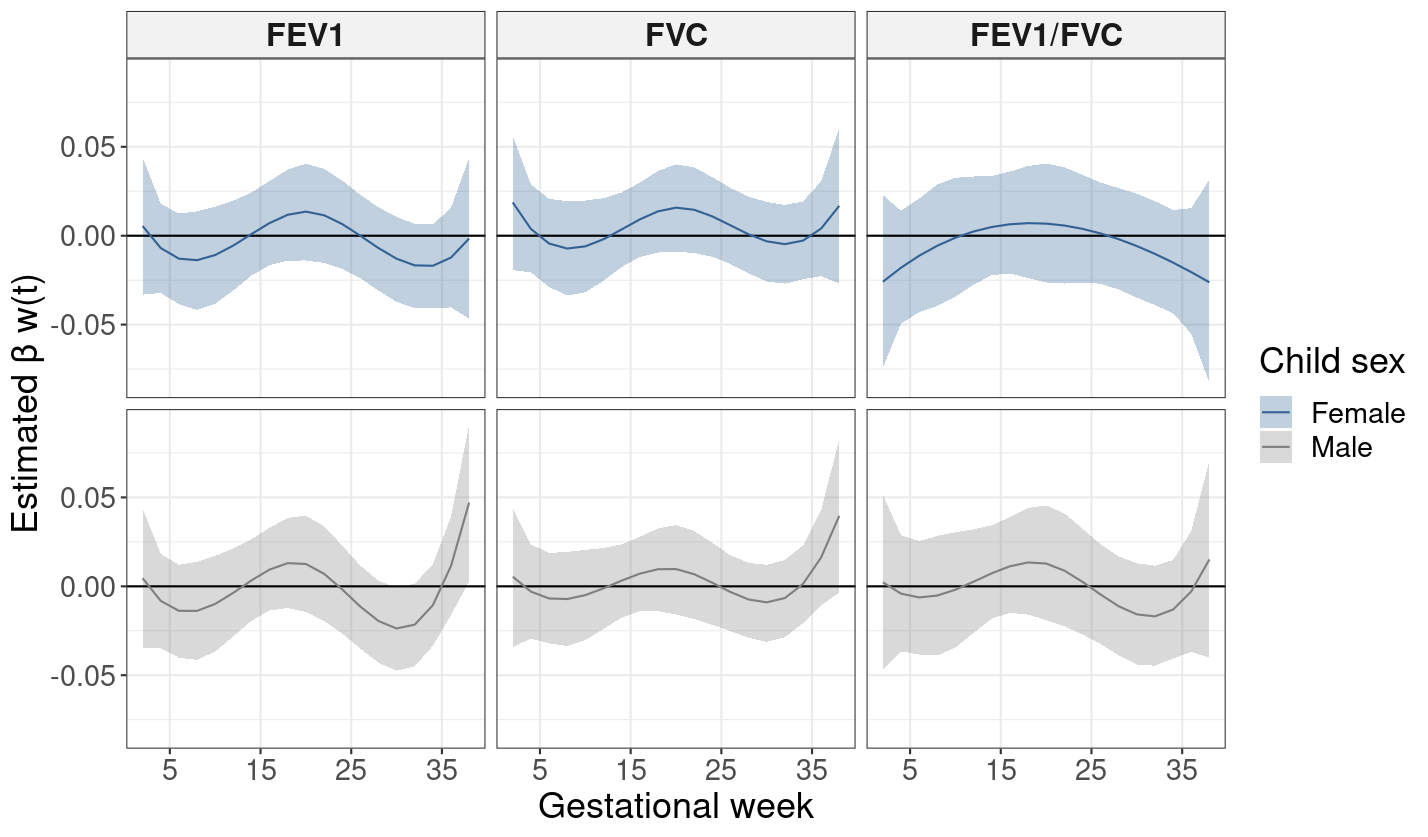
Figure S2. Associations between prenatal PM_2.5_ and child lung function (per 2-µg/m^3^ increase in PM_2.5_ during a given two-week period) among children with and without allergic sensitization (food or aeroallergen IgE levels >= 0.35 kU/L) in BDLIM models. For FEV1, the model with the highest posterior predictive density was one in which only the effect size *β* and weight function *w(t)* varied by child sex. For FVC, the model with the highest posterior predictive density was one in which neither the effect size *β* or weight function *w(t)* varied by child sex. For FEV1/FVC, the model with the highest posterior predictive density was one in which only the time-varying varied by child sex.
